# USA’s expanded overseas tuberculosis screening program: a retrospective study in China

**DOI:** 10.1186/s12889-015-1558-z

**Published:** 2015-03-07

**Authors:** Shaojun Liang, Jianming Zhang, Longfei Hu, Jiandong Chen, Jian Wu, Yongxin Huang, Yan Zeng, Yufeng Zhu, Zhaohui Li, Ying Wen, Wuyi Liang, Jinxue Zhuo, Hongtao He

**Affiliations:** Guangdong International Travel Healthcare Center, 5/F, Eastern Tower, Poly Building, 59 Huali Road, Zhujiang New City, Guangzhou, Guangdong 510600 PR China; Shenzhen Entery-exit Inspection and Quarantine Bureau, 1101 Fuqiang Road, Futian District, Shenzhen, Guangdong 518045 PR China

**Keywords:** Benefit, Immigrant, Overseas, Screening, Tuberculosis, USA, China

## Abstract

**Background:**

To address increasing tuberculosis (TB) incidence in foreign-born populations, immigrant TB screening programs have been implemented in the USA. These programs are modified periodically, the effectiveness of which have been disputed. The aim of this retrospective study was to assess the value of the 2009 Technical Instructions for Tuberculosis Screening and Treatment Using Cultures and Directly Observed Therapy (CDOT TB TI) in a cohort of the USA permanent-resident applicants from China.

**Methods:**

Standardized forms were used to collect demographic, clinical, and laboratory data of Chinese individuals screened at the Guangdong International Travel Healthcare Center for permanent residence in the USA between October 08, 2009 and December 31, 2012. Applicants’ data were further retrospectively evaluated by three experienced panel physicians and radiologists according to the 1991 Technical Instructions for Tuberculosis Screening and Treatment (TI). TB cases and characteristics identified by the 1991 and expanded 2009 programs were compared.

**Results:**

The CDOT TB TI identified more than twice as many TB cases that required treatment completion before clearance for travel than the 1991 TI (270 *vs.* 131). In addition, the expanded screening program identified more cases of negative sputum smear but positive culture (181 *vs.* 44), and more cases of radiography suggestive of inactive (22 *vs.* 3) and active (248 *vs.* 128) TB. Specifically, the 1991 TI screening program failed to identify 25/38 (65.79%) cases carrying drug-resistant isolates, and 13/131 (9.92%) would have been inappropriately treated. Moreover, 220/270 (81.48%) of the cases were asymptomatic, which were identified by screening and subsequently treated. Improved chest radiograph and sputum negative conversion occurred in all treated cases.

**Conclusion:**

CDOT TB TI, a screening program that includes sputum culture and drug susceptibility tests, identifies a greater number of TB cases, likely contributing to the overall decrease in TB prevalence in host (USA) and origin (China) countries.

## Background

For industrialized countries with a low burden of tuberculosis (TB), the incidence of the disease is primarily attributed to foreign-born individuals originating from high-TB burden countries [1-8]. To combat this, programs for TB screening among immigrants have been instituted and evaluated [9,10]. Typically, screening involves chest radiograph (CXR) alone or with clinical examination, though sputum tests have recently been included [1,11]. However, these programs vary with regard to national legislation, resource availability, and public health risk management practices [1,11-13], and their coverage and effectiveness remain disputable [14,15].

As the country with the largest number of international migrants, the percentage of emigrant TB cases in the USA has increased substantially since the Centers for Disease Control and Prevention (CDC) began collecting data on place of birth in 1986 [16-19]. During this time, technical instruction and guidance for TB screening has periodically been modified [11,20,21]. Beginning in 1991, the CDC Technical Instructions for Tuberculosis Screening and Treatment (TI) required that individuals overseas applying for permanent residence in the USA undergo TB screening. For those suspected of having active pulmonary TB, laboratory investigation was required, but not sputum culture or drug susceptibility testing (DST) [18,21-23]. In 2007, these requirements were expanded to include sputum cultures for applicants with signs or symptoms of TB, an abnormal CXR suggestive of TB disease, or human immunodeficiency virus (HIV) infection. The updated version in 2009 was named as the TI Using Cultures and Directly Observed Therapy (CDOT TB TI). Additional revisions incorporated DST for positive cultures to guide treatment and a shorter validity period for travel clearance [22,23]. As a result, the expanded overseas screening protocol reduced the importation of active TB disease from Mexico, the Philippines, and Vietnam [20], resulting in an annual savings of $15 million [24]. However, data from the countries of origin are needed to obtain definitive conclusions concerning the effect of CDOT TB TI on reduced importation of TB.

China has become the 4th largest source of migrants in the world [25]. China is also ranked 4th of the top 5 source countries of TB-infected emigrants to the USA, and a large number of USA-bound immigrants with suspected TB are identified in overseas screening [19,20]. Although the expanded screening program was implemented in China in 2009, there are no current reports evaluating the effects of CDOT TB TI on TB epidemiology in USA-bound migrants or within China or other countries of origin. Therefore, we studied TB cases among applicants for permanent USA residence at the largest assigned panel site in China, the Guangdong International Travel Healthcare Center (GDITHC) of Guangdong Province. Specifically, we aimed to evaluate the effectiveness of the CDOT TB TI and identify factors that may aid other emigration and immigration countries with respect to TB incidence.

## Methods

### Study design and applicants

A retrospective study was conducted to assess the effectiveness of the expanded screening program (CDOT TB TI) on individuals applying for permanent USA residence at GDITHC between October 08, 2009 and December 31, 2012. In accordance with the CDOT TB TI, Chinese applicants ≥15 years of age require a medical history, physical examination and CXR; applicants 2–15 years of age require a medical history, physical examination and tuberculin skin test or interferon-gamma release assay and should undergo CXR evaluation if a tuberculin skin test ≥ 10 mm or interferon-gamma release assay is positive. Applicants with signs or symptoms of TB disease, an abnormal CXR suggestive of TB disease, or HIV infection are required to provide three consecutive morning sputum specimens. Sputum tests include a smear for acid-fast bacilli (AFB) and culture for *Mycobacterium tuberculosis* (MTB).

Applicants’ data were further retrospectively evaluated according to the 1991 TI for comparison. For these evaluations, three experienced panel physicians and radiologists were invited to review related CXRs and other clinical characteristics, and to provide a unanimous final decision.

Applicants with infectious pulmonary TB disease, including laryngeal and pleural TB, must undergo directly observed therapy at a CDC Division of Global Migration and Quarantine (DGMQ)-approved treatment site before travel is allowed. For those treated at non-DGMQ-approved sites, the evaluation must be repeated one year after completion of treatment prior to travel clearance. All treatment regimens are required to follow guidelines of the current American Thoracic Society/CDC/Infectious Diseases Society of America, which incorporate DST results.

### Data collection

Standardized forms were used to collect applicants’ demographic, clinical, and laboratory data from the working and surveillance database of GDITHC, including: date of birth, sex, medical history (including prior history of TB disease and treatment), physical examination findings, TB exposure, current TB symptoms and signs, HIV-test results, CXR findings, AFB sputum smear and MTB culture results, and treatment course and outcomes (up until March 31, 2014). To compare epidemiologic characteristics of TB among the applicants, Guangdong Province, and China as a whole, epidemiologic data were cited from two published articles describing the results of the Fifth National Survey on TB Disease conducted in 2010 [26,27].

### Statistical analysis

All analyses were performed using SPSS 14.0 software (SPSS Inc., Chicago, IL, USA). A *χ*^*2*^ or Fisher’s exact test was used to compare proportions. Two-tailed *P* < 0.05 was considered as statistically significant.

### Ethical review

This study was exempt from approval by the GDITHC Review Board as it analyzed working and surveillance data that is routinely collected, and applicant-identifying information was removed.

## Results

**Figure 1:**
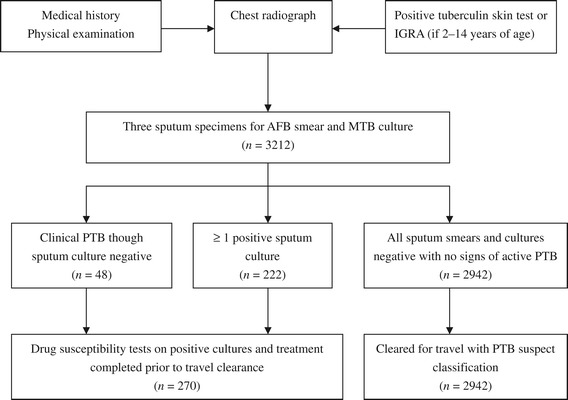
**Summary of overseas tuberculosis screening algorithm based on the Technical Instructions for Tuberculosis Screening and Treatment Using Cultures and Directly Observed Therapy.** AFB = acid-fast bacilli, IGRA = interferon-gamma release assays, MTB = *Mycobacterium tuberculosis*, PTB = pulmonary tuberculosis, TB = tuberculosis.

In total, 83,214 applicants for USA permanent residence underwent overseas TB screening at GDITHC during the study period; the male to female ratio was 1.87/1 and mean age was 39 y (range: 3 mo to 87 y). Approximately half of the applicants came from Guangdong Province where the panel site is located. A summary of the applicant screening process is presented in Figure 1. Of the 3,276 applicants with abnormal CXR suggestive of TB disease, either active or inactive, and one with a positive HIV status, 3,212 completed the screening procedures; 65 applicants failed to provide the required sputum specimens. Screening results indicated that 270 cases were required to complete TB treatment before clearance for travel was provided, for a TB rate of 0.32% (270/83,214).

Characteristics of the identified TB cases are presented in Table 1. Similar to the findings in Guangdong Province [26,27], 58.52% (158/270) of the TB cases were among individuals 35–64 years of age. Twelve applicants had been diagnosed with active pulmonary TB by the National Tuberculosis Control Program (NTP) as they reported symptoms of chronic cough (cough ≥ 2 wk), bloody sputum, or chest pain, and were still taking TB medicines at the time of screening, whose sputum tests came back as negative for both smear and culture. A total of 38 applicants failed to get treatment for chronic cough, fatigue, and low fever before screening, from whom 35 MTB isolates were detected and three sputum tests came back as negative for both smear and culture. A large majority (81.48%; 220/270) of the identified cases had no current TB symptoms or treatment, with or without history of previous TB disease/treatment, much higher than in National survey (43.1%) [27]; 187 of these 220 individuals were MTB-culture positive, 6 were nontuberculous mycobacteria (five with positive-AFB smear), 10 were negative for culture but had positive-AFB smears, and the remaining 17 were negative for smear and culture. All 20 of the individuals with negative smears and cultures (three reporting current TB symptoms without treatment and 17 without symptoms or treatment) were clinically diagnosed with smear-negative pulmonary TB, which was consistent with the definition in the International Standards for Tuberculosis Care of the World Health Organization: positive tuberculin skin test or interferon-gamma release assays, chest radiography suggestive of active TB, no improvement in response to a course of broad-spectrum antibiotics (excluding anti-TB drugs, fluoroquinolones, and aminoglycosides), but improvement in response to 1–2 months of a standard anti-TB regimen consisting of isoniazid, rifampicin, pyrazinamide and ethambutol. According to the CDOT TB TI, the clinically diagnosed smear-negative cases and those with positive-AFB smear and nontuberculous mycobacteria were all required to complete TB treatment before clearance for travel. Among the 32 cases with negative sputum smears and cultures, 12 (37.6%) were currently on TB medications, and 20 (62.5%) were required to complete TB treatment due to clinical diagnosis of smear-negative pulmonary TB. The rate of positive AFB smears among applicants (0.06%) was not different from that of Guangdong Province (0.04%) or the National Survey on TB Disease (0.07%), whereas the rate of bacterial-positive pulmonary TB was significantly higher (0.27 *vs.* 0.06 and 0.14%, respectively; *P*s < 0.05) [26,27]. Among the 222 MTB isolates, 17.12% (38/222) showed first-line drug resistance and 2.25% (5/222) were multidrug resistant (MDR), most commonly to isoniazid and streptomycin, followed by rifampicin. Primary drug-resistant isolates were detected in 16.67% (33/198) of applicants, which is similar to the 18.4% reported in Guangdong Province [26,27].

**Table 1:** **Characteristics of TB cases identified under different technical instruction**

**Characteristics**	**CDOT TB TI (** ***n*** **)**	**1991 TI (** ***n*** **)**
TB cases identified	270	131
Male sex	176	88
Age group, y		
≤24	26	15
25–34	37	20
35–44	35	16
45–54	60	26
55–64	63	30
65–74	33	20
≥75	16	4
Medical history investigation		
TB now, on treatment	12	12
TB previous, treated	33	20
TB previous, untreated	8	0
TB previous suspect classification, untreated	5	2
Current TB symptoms	40^§^	19
Chest radiograph characteristics		
Consistent with inactive TB	22	3
Consistent with active TB	248	128
Sputum test		
Positive AFB smear and MTB culture	41	40
Positive AFB smear and negative MTB culture	10	10
Negative AFB smear and positive MTB culture	181	44
Negative AFB smear and negative MTB culture	32	31
Nontuberculous mycobacteria	6^*^	6
Drug susceptibility test for MTB isolates		
Total isolates	222	84
Pan-susceptible to first-line anti-TB drugs	184	71
First-line primary-drug resistant	33	11
First-line acquired-drug resistant	5	2
Multidrug resistant	5^¶^	2

Abbreviations: *AFB* = acid fast bacilli, *CDOT TB TI* Technical Instructions for Tuberculosis Screening and Treatment Using Cultures and Directly Observed Therapy (2009), *MTB* = *Mycobacterium tuberculosis,* *TB* = tuberculosis, 1991 *TI* = Technical Instructions for Tuberculosis Screening and Treatment (1991).

Notes: ^§^two persons were currently on treatment; *one with negative AFB smear, ^¶^four cases of primary and one case of acquired multidrug resistance.

**Figure 2:**
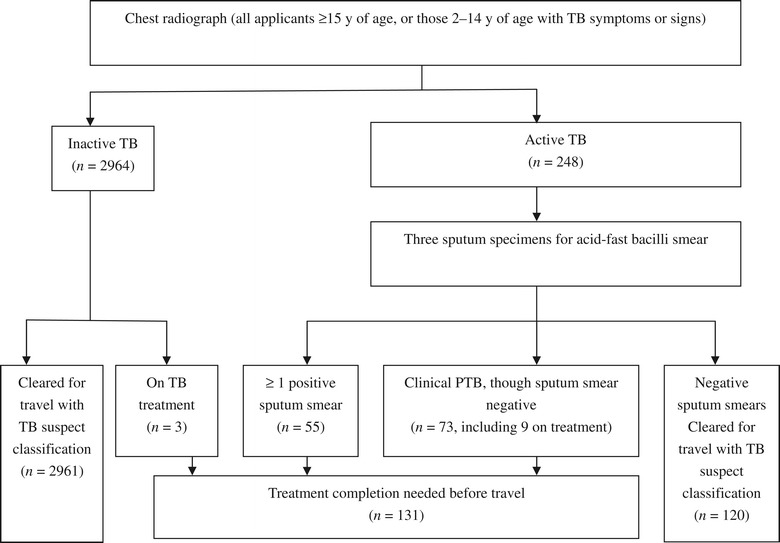
**Summary of overseas tuberculosis screening algorithm based on the Technical Instruction for Tuberculosis Screening and Treatment (1991).** PTB = pulmonary tuberculosis, TB = tuberculosis.

All the applicants’ data were further retrospectively reviewed according to the 1991 TI protocol, for which 248/83,214 applicants would have been required to submit sputum specimens based on CXR suggestive of active pulmonary TB (Figure 2). Of these, 55 applicants would have been identified with infectious active pulmonary TB (due to ≥ 1 positive sputum smear), and an additional 73 applicants would not have been cleared for travel because of clinical diagnosis warranting treatment for pulmonary TB regardless of laboratory results; 9/73 had been on TB medications at the time of screening and 64/73 would have been diagnosed as smear-negative pulmonary TB. Actually, 44 of the 64 individuals that were negative for smear were positive for MTB when culture testing was performed. The 1991 TI would have also required three cases with CXR suggestive of inactive TB but still on TB medications to complete treatment before travel clearance would be granted. Thus, only 0.16% (131/83,214) of applicants would have been retained from clearance for travel, a rate that is roughly half of the result based on the CDOT TB TI. As shown in Table 1, using the older TI guidelines, nearly half of the applicants with CXR suggestive of active TB would not have been restricted from travel due to negative AFB smears. Furthermore, a significantly smaller proportion of applicants with CXR suggestive of inactive TB would have been detected (2.3 *vs.* 8.1% by CDOT TB TI; *P* < 0.05). Moreover, approximately two-thirds of the cases carrying drug-resistant isolates would have been missed based on the 1991 TI, including three MDR-TB cases. Among the 131 cases required to complete TB treatment, 9.92% (13/131) carrying drug-resistant isolates would most likely have been treated with a six-month standard TB regimen, which most likely would have failed due to lack of DST, including two MDR-TB cases.

Of the identified applicants, 227 completed their TB treatment, which lasted 6–12 mo (24 mo for the two MDR-TB cases). Culture negative conversion within 60 d was slightly higher for applicants treated at the DGMQ-approved sites compared with non-approved sites (97.5 *vs.* 94.5%). Two MDR-TB cases completed their directly observed therapy consisting of second-line drugs at GDITHC, while the other three cases were lost. After completion of treatment, CXR revealed improvements in lung lesions in 227 applicants, including the 22 cases with initial CXR findings suggestive of inactive TB. Only one case relapsed during the study period, involving an applicant who had completed a treatment course at a non-approved treatment site, and had been waiting for another year. Eight applicants were unable to emigrate to the USA after completing treatment as the validity period for travel clearance was too short (3 mo), despite the absence of positive culture conversion.

## Discussion

Previous studies have indicated that the inclusion of the MTB culture would identify an additional 250 TB cases per year in foreign-born individuals screened prior to USA entry [21,28]. In fact, the number of positive-culture TB cases detected among recent USA immigrant arrivals has dramatically decreased after implementing the expanded screening protocol [20]. Consistent with this, the results of this study indicate the expanded overseas screening program prevented the emigration of more than double the TB cases from China, primarily from cases with negative AFB smears. Although such cases are less infectious, the prevalence of negative-smear TB cases among immigrants and refugees highlights the necessity for effective screening and management to protect public health [29].

The highest rates of TB cases occur near to the time of entering among foreign-born individuals in the USA [28], similar to other countries with a low TB epidemiology [30]. Although the TB incidence typically drops among these immigrants after one or two years, foreign-born individuals are still more likely develop TB disease than native-born individuals, even after 20 years [28,31,32]. This is attributed to latent TB infection (LTBI), which presents an important and cost-effective opportunity for eliminating TB in host countries [5,6,8]. However, these studies may have included individuals who were considered free of active TB, or even had CXR suggestive of active TB, but may have had positive cultures if sputum tests were conducted. These cases then enter the host country with a TB suspect classification, which can be considered as LTBI, and would thus be diagnosed during subsequent domestic follow-ups. Between 63.6% and < 100% of these immigrants completed follow-up evaluations [29], as it is voluntary for individuals with suspect TB classification to visit a health department after entering the USA [18], and detection of missed cases is prolonged after arrival. This may contribute to the high TB incidence within the foreign-born population, even decades after entry.

The use of CXR-based TB screening protocols in foreign-born populations has been disputed in many countries [33-35]. In CDOT TB TI, CXR screening further evaluates cases with results indicating inactive pulmonary TB. The CDOT TB TI also stipulates that a greater number of these applicants undergo an MTB test (e.g. 3,212 *vs.* 248 with 1991 TI based on our findings). Moreover, inclusion of the sputum culture in the expanded TB screening program doubled the number of active TB cases that required completion of treatment before being allowed to emigrate from China to the USA.

More asymptomatic TB cases were detected at GDITHC than reported in the National Survey in 2010 [26,27]. An explanation for this is that immigration health screening represents an active case-finding method and identifies cases at an earlier stage of disease. Consequently, immediate treatment of these cases prevents the transmission of MTB within the surrounding population. The expanded CDOT TB TI provided early diagnosis and treatment for 258 applicants in this study. Importantly, 85.0% of the asymptomatic TB cases in this study were confirmed by bacterial culture, highlighting the value of the sputum test. In China and many developing countries, there is an insufficient number of capacity-qualified labs [36]. Thus, panel sites that follow the CDOT TB TI and qualify for smear microscopy and culture and DST capacity can benefit the NTP of countries of origin by identifying and treating more cases [24].

Many of the TB applicants in this study ignored TB symptoms, similar to findings in the National Survey in China in 2000 and 2010 [26,37]. The mildness of the symptoms, along with insufficient knowledge and attitude concerning TB, likely contributed to the failure to seek treatment. Once arriving in the host country, unfamiliarity with the healthcare system and fear of being repatriated can further delay medical evaluation [38]. Therefore, health screening of permanent residence applicants provides an opportunity for education and promotion of healthcare-related topics [9,13].

Several studies have questioned the quality of work performed at panel sites [11,18,21,39] despite the documented value of overseas screening [29,40]. The 0.3% TB detection rate at GDITHC was similar with other studies [11,13], though much lower than that in Vietnam (9.4%) [21]. Indeed, the prevalence of TB has remarkably declined in China in recent years, only 459/100,000 in 2010 and 230/100,000 in Guangdong Province [26,27], largely because of a great effort from the NTP [36]. A large proportion of applicants from our study were from Guangdong Province, thus, the 0.3% TB detection rate at GDITHC is reasonable and consistent with that of Guangdong Province and lower than national data. The age distribution and proportion of primary drug-resistant isolates of this study cohort were also similar to the survey in Guangdong.

The obvious disadvantage of the CDOT TB TI is that many applicants with CXR findings consistent with “old healed TB,” including those who do not need further treatment, are required to undergo a sputum test before being cleared for travel. This requirement imposes a lengthy delay, as results of sputum cultures can take 42 days, compared to obtaining results from the sputum smear test in one day. However, information related to prolonged wait times or the additional expense is not routinely collected during pre-visa medical evaluations. Extensive study should be carried out on this important topic of cost-effectiveness of the expanded overseas TB screening policy.

There are a few limitations of this study worth mentioning. First, the study cohort was entirely comprised of applicants at the GDITHC, one of the panel sites in China; thus, selection bias may play a role in this study. Second, due to lack of follow-up data of the studied group of applicants after arriving in the USA, the long-term implications of TB prevalence in the USA cannot be determined.

## Conclusion

Compared to the 1991 TI, the CDOT TB TI identified a larger number of TB cases that require treatment prior to emigration, including asymptomatic cases and cases missed by the NTP. The inclusion of MTB cultures played a key role in finding cases that can be considered LTBI, which will likely impact results from domestic follow-ups. Overseas panel sites following CDOT TB TI can identify and treat more asymptomatic cases, serving as a useful supplement to the NTP. As global investment is the ideal long-term TB control strategy [24,41], programs such as the CDOT TB TI represent a viable, effective management system, as evidenced by the reduced prevalence of TB in China.
